# Matching the Material of Transparent Objects: The Role of Background Distortions

**DOI:** 10.1177/2041669516669616

**Published:** 2016-09-12

**Authors:** Nick Schlüter, Franz Faul

**Affiliations:** Institut für Psychologie, Christian-Albrechts-Universität zu Kiel, Germany

**Keywords:** material perception, perceptual transparency, optical background distortion, refractive index, material matching

## Abstract

It has been proposed that the visual system is able to estimate the refractive index of thick transparent objects from background distortions caused by them. More specifically, it was hypothesized that this is done based on a mid-level cue, the distortion field, whose computation from the input requires comparing the part of the background seen through the object with the part visible in plain view. We test two predictions derived from this hypothesis: (a) scene variables that do not change the distortion field, for instance, the density of the background texture, should not systematically influence the subjects’ settings in a material matching task. (b) The uncertainty of the estimates should increase sharply, if the part of the background texture in plain view is removed. Our results are not compatible with these two predictions but are completely in line with the alternative interpretation that the subjects maximized the similarity of the distorted background textures on the image level. Additional results indicate that subjects can take relations between the distorted and the undistorted background into account if this is encouraged by the experimental design, but they do this in a simplistic way that is inappropriate to estimate the refractive index.

## Introduction

**Figure 1.**
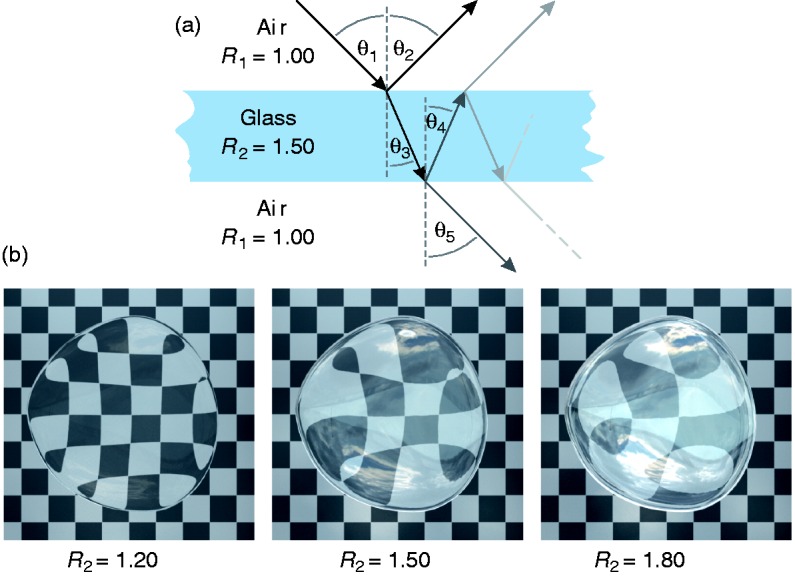
Refraction and reflection and their effects on the appearance of light-transmitting objects. (a) If light hits a light-transmitting object whose material has a higher refractive index (*R*) than that of the surround, for example, a pane of glass surrounded by air, it is partly reflected (with the angle of reflection being equal to the angle of incidence, i.e., θ_2_ = θ_1_) and partly refracted toward the surface normal (i.e., θ_3_ < θ_1_). If the refracted light hits the opposite boundary of the object, it again splits up. However, the refraction occurs in the opposite direction (i.e., θ_5_ > θ_3_) and higher angles of incidence can even lead to total reflection. (b) Effects of refraction and reflection on the retinal projection of a thick irregularly shaped light-transmitting object without absorption or subsurface scattering. The strength of specular reflection determines the brightness of the mirror image of the environment on the object’s surface. The strength of refraction determines the degree of background distortion. Both the specular reflection and the amount of refraction increase with the refractive index. Note that the shape of the object is the same for all three images.

Light-transmitting materials often appear transparent. Previous investigations of perceived transparency that relate to such materials have mainly focussed on their transmission properties and correspondingly considered the color and luminance relations that lead to perceptual transparency (Beck, 1978; Beck, Prazdny, & Ivry, 1984; Faul & Ekroll, 2002, 2011; Khang & Zaidi, 2002a, 2002b; Ripamonti, Westland, & Da Pos, 2004; Robilotto, Khang, & Zaidi, 2002). These investigations used highly reduced situations in which simple flat filters without refraction and scattering were viewed frontally under a homogeneous illumination. Recent progress in computer graphics made it possible to simulate more complex light-transmitting objects. This includes translucent objects like wax or milk, which due to subsurface scattering appear more or less hazy. Fleming and Bülthoff (2005) and Motoyoshi (2010) proposed specific cues that can be used to identify such objects and to characterize their properties. A further class of objects that can now be investigated are clear light-transmitting objects that are made of refractive materials but lack absorption and scattering. Light incident on the surface of a refractive material is partly reflected and partly transmitted (Figure 1(a)). The relative amount of these two parts can be predicted by Fresnel’s equations and depends on the refractive index *R* of the object’s material and the angle of incidence θ. The reflected light leaves the surface in the mirror direction (specular reflection). The transmitted light is bend at the surface by an angle that is given by Snell’s law (refraction). The effects of refraction and specular reflection on the retinal projection of light-transmitting objects are especially apparent for thick, irregularly shaped objects like the ones shown in Figure 1(b).

Due to the specular reflections, the environment is partly visible at the object’s surface. This suggests that previous investigations dealing with the perception of opaque mirror surfaces that specularly reflect all incoming light (Blake & Bülthoff, 1990, 1991; Fleming, Torralba, & Adelson, 2004; Savarese, Chen, & Perona, 2004, 2005; Savarese, Fei-Fei, & Perona, 2004) are of some relevance. However, it is at present unclear to what extent these findings can directly be transferred to light-transmitting objects, which show a more complex reflection behavior. In these objects, the amount of reflection depends not only on the material’s refractive index but also on the viewing angle: It is low at parts of the surface where the viewing direction is nearly perpendicular and approaches 1 at grazing angles. Furthermore, multiple specular reflections that originate from different surfaces, for instance, the frontal and the back plane of a pane of glass, can be superimposed in the image. This is because specular reflections occur at each boundary of a light-transmitting object, irrespective of whether the light hits the boundary from the inside or the outside of the object.

The refraction, which is of primary importance to the present investigation, leads to distortions in the image. These are especially pronounced for thick irregularly shaped light-transmitting objects, like the blob of clear glass shown in Figure 1(b). Two recent studies proposed that these background distortions are used by the visual system to infer object properties. Chen and Allison (2013) considered the relationship between optical distortions and object shape. Their results suggest that the pattern of distortions is actually used as a cue for shape. Fleming, Jäkel, and Maloney (2011) referred to the regular relationship between optical distortions and the refractive index of the object’s material: The larger the difference in the refractive indices at the interface between two media, the larger the distortions. They tested the hypothesis that the visual system uses optical distortions to estimate the refractive index (RI hypothesis). In their matching experiment, two thick transparent objects were presented in different static scenes, and the subjects’ task was to adjust the refractive index of the test object until it appeared to be made of the same material as the standard object. They found a high correlation between the refractive indices set by the subjects and the fixed refractive indices given in the standard objects. This led the authors to conclude that “the pattern of image distortions that occurs when a textured background is visible through a refractive object provides a key source of information that the brain can use to estimate an object’s intrinsic material properties” (p. 818).

Testing the RI hypothesis in this direct way is highly problematic for two reasons. A first problem is confounding variables. Physically correct stimuli as the ones used by Fleming et al. (2011) contain not only background distortions but also specular reflections, which likewise depend on the refractive index. In Schlüter and Faul (2014), we have shown that specular reflections can substantially influence the subjects’ settings in such matching tasks. It is thus difficult to discern the relative influence of background distortions and specular reflections on the settings. Simply removing specular reflections from the stimulus to isolate the influence of distortion information does not solve this issue because this in turn strongly reduces the impression of transparency (see Experiment 1b in this article). A second problem is how systematic deviations from a perfect match, as they were found in the experiments of Fleming et al. (2011), should be interpreted. While such deviations could be attributed to the heuristic nature of visual perception or unfavorable stimulus conditions, they could as well indicate a fundamental flaw in the underlying theory. The interpretation of such deviations is especially difficult because the RI hypothesis—at least in its current form—is not specific enough to quantitatively predict the susceptibility of the estimates to variations in the input. It seems, therefore, necessary, to evaluate the RI hypothesis in a more indirect way that encompasses systematic tests of hypotheses derived from the theoretical assumptions, critical comparisons of the experimental results with the predictions of alternative explanations, and theoretical analyses of computational and functional aspects. As a first step in this direction, we presented in Schlüter and Faul (2014) a theoretical analysis that leads to the conclusion that the RI hypothesis is implausible from a computational perspective. Furthermore, we found that the subjects’ settings in the matching task were substantially influenced by irrelevant context factors.

**Figure 2.**
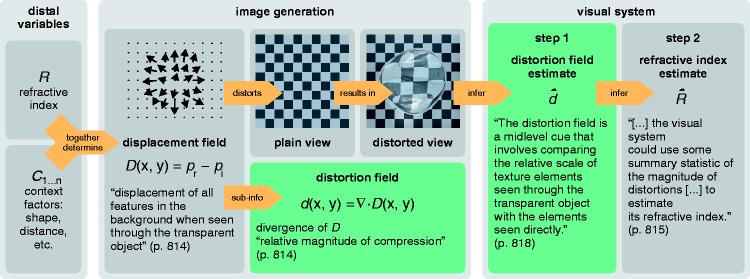
Schematic illustration of the RI hypothesis of Fleming et al. (2011), which states that the visual system can use image distortions caused by thick transparent objects to estimate the refractive index of the object’s material. The authors describe the distortions by means of a 2D vector field, the displacement field *D*, that contains information about the direction and magnitude by which the position of each image feature is displaced in the distorted view (*p*_r_) compared with the plain view (*p*_i_). The displacement field depends on the refractive index of the material (*R*) and several context factors (*C*_1 … n_, e.g., object shape, background distance, viewpoint). Fleming et al. (2011) suggest that the visual system refers to the divergence *d* of the displacement field, which represents only part of the information available in *D*. They assume that this so-called distortion field *d* can be estimated by comparing the relative scale of texture elements within the object and the surround (Step 1). This distortion field estimate $\overset{\wedge }{d}$ is then supposed to be used to infer the refractive index estimate $\overset{\wedge }{R}$ (Step 2).

In the present article, we test two predictions derived from the computational ideas that Fleming et al. (2011) presented as a specification of their RI hypothesis (see Figure 2 for a schematic illustration). They first consider the *displacement field D*, a 2D vector field that “measures the displacement of all features in the background when seen through the transparent object” (p. 814): *D*(*x*, *y*) = *p*_r_ − *p*_i_, with *p*_i_* *= (*x*, *y*) being the image location of a feature in undistorted plain view and *p*_r_ = (*x*′, *y*′) being the location of the same feature when refracted by the transparent object. Second, they consider the divergence *d*(*x*, *y*) = ▽ · *D*(*x*, *y*) of the vector field *D*, which they call the *distortion field.* The divergence is a scalar field that represents only a part of the information contained in the displacement field *D.* Its values are related to the “relative magnitude of compression” (p. 814) of the texture pattern. The core assumption of Fleming et al. (2011) is that it is somehow possible to compute an estimate $\overset{\wedge }{d}$ of the distortion field *d* from the input image and that this estimate could then be used in a second step to infer the refractive index. If both *D* and *p*_i_ are completely unknown, it is obviously impossible to estimate $\overset{\wedge }{d}$ from the information given in the distorted image *p*_r_ alone. This implies that in order for this computational idea to work, constraints for *p*_i_ are required. Fleming et al. (2011) suggest that these constraints can be obtained by referring to the undistorted background, if it is visible in the surround of the object. More specifically, they propose that using the distortion field as a cue “involves comparing the relative scale of texture elements seen through the transparent object with the elements seen directly” (p. 818). In addition to the problems associated with estimating the distortion field, Fleming et al. (2011) identify the further problem of “how the local estimates of distortion magnitude are pooled into a global estimate of RI” (p. 818). In our previous investigation of the RI hypothesis (Schlüter & Faul, 2014), we mainly focused on this RI estimation problem (i.e., step 2 in Figure 2) and argued that it would be computationally highly complex because of the many context factors that also influence the distortions but are not linked to the object’s refractive index (e.g., the viewpoint, the object’s shape or the distance of the background).

In this article, we concentrate on empirical tests of the first step, the estimation of the distortion field. To this end, we derive two testable predictions from the RI hypothesis that do not require any knowledge about the second step that presumably uses the estimate $\overset{\wedge }{d}$ to infer the object’s refractive index. In essence, the first expectation is that the subjects’ settings should be largely invariant against global changes in the background texture because this manipulation neither systematically influences the distortion field nor the relative scale of the texture elements in the distorted and undistorted parts of the background. A second expectation is that the error in the subjects’ settings should increase substantially if the putatively important information given in the object’s surround is removed because the assumed computational strategy is then no longer possible.

We test both predictions and compare them with the predictions derived from the alternative explanation presented in Schlüter and Faul (2014), which assumes that the subjects do not match estimated refractive indices but instead simply maximize the similarity of the presented stimuli on the image level. This matching strategy is completely unrelated to transparency perception.

## Experiment 1: The Influence of Background Texture Density

Experiment 1 tests the first prediction derived from the RI hypothesis that changes in scene variables that do not influence the displacement field should have no systematic effect on the refractive index settings. This test is described in Experiment 1a. In Experiment 1b, we compare the results of Experiment 1a with the predictions from our alternative explanation.

### Experiment 1a: Testing the Prediction of the RI Hypothesis

The displacement field *D* and thus also the distortion field *d* depend on several variables, for example, the material and the shape of the object, its distance to the background, the viewing angle, *but not* on the background texture. In a scene that is geometrically fixed, *D* may be regarded as an operator that acts on arbitrary backgrounds and in each case yields the same distorted version of it. Thus, if the RI hypothesis is correct, then the refractive index estimate should be invariant against global changes in the background texture, as long as the texture contains enough structure to reflect the optical distortions truthfully.

**Figure 3.**
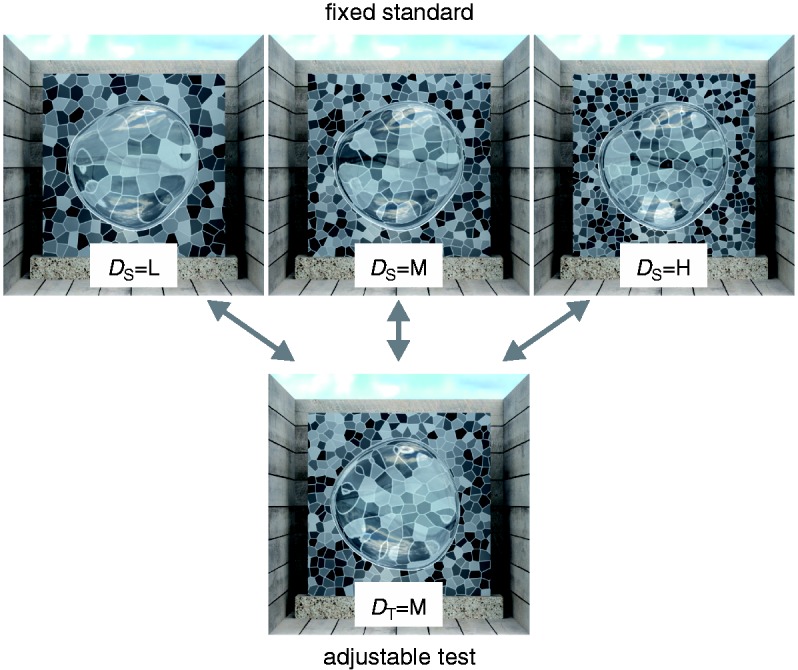
In the material matching task of Experiment 1a, a fixed standard stimulus, whose background texture density and refractive index varied according to the experimental condition, was compared with a test stimulus, whose refractive index was adjustable by the subjects. The texture density of the test stimuli (*D*_T_) was medium (“M”) throughout the experiment. The density of the standard stimuli (*D*_S_) was identical (“M”), higher (“H”), or lower (“L”) than in the test. The objects illustrated here have identical refractive indices (*R*_S_ = *R*_T_ = 1.5).

To test this prediction, we performed an experiment similar to the ones conducted by Fleming et al. (2011) and Schlüter and Faul (2014). The subjects saw two transparent objects of slightly different shape that were placed in front of backgrounds with different texture densities. All other scene variables were identical. The task was to match the material properties of a standard and a test object by adjusting the refractive index of the test (see Figure 3).

#### Stimuli

The stimulus material consisted of computer-generated images that were created with the Mitsuba renderer (Jakob, 2013). They were similar to the ones used in Schlüter and Faul (2014), which were designed to closely resemble the ones used by Fleming et al. (2011). The stimulus images showed a thick transparent object in front of a background plane that was located inside a box with front and top sides open (cf. Figure 3). All scene elements were defined in real-world coordinates relative to a virtual projection plane, which represented the surface of the experimental screen. Standard and test objects were two slightly different warped ellipsoids. The objects were modeled with the 3D computer graphics software Blender (Blender Foundation, 2013), by applying various shape modifiers to an icosahedron that was subdivided eight times. The objects were located at the center of the virtual projection plane, had a size of 50 × 50 × 6 mm and were defined to be light-transmitting (“dielectric”) without any absorption. The refractive index of the standard object varied in three steps (*R*_S_ ∈ {1.20, 1.50, 1.80}). The refractive index of the test object was adjusted by the subjects (*R*_T_ ∈ {1.010, 1.015, … , 2.495, 2.500}, 299 steps). The refractive index of the surrounding medium was set to 1.

The textured background plane (80 × 80 mm) was placed behind the transparent object at a distance of 60 mm. The background textures were random Voronoi patterns created with Matlab (The MathWorks, 2013) that resembled the background textures used by Fleming et al. (2011). The individual faces of the pattern were separated by bright achromatic (*x* = .30, *y* = .32, *L* = 52.75 cd/m^2^) seams of .32 mm width. The faces were also achromatic (*x* = .31, *y* = .32), and their luminances were uniformly distributed between 4.43 and 47.43 cd/m^2^. The density of the background textures of the standard stimuli varied in three steps according to the experimental condition (*D*_S_ ∈ {#x0201C;L”, “M”, “H”}, see Figure 3). The medium density texture (“M”) contained roughly 20 × 20 faces, the low density texture (“L”) 15 × 15 faces and the high density texture (“H”) 25 × 25 faces. The density of the background texture for the test stimuli was the same throughout the experiment (*D*_T_ = “M”, roughly 20 × 20 faces). It was computed with a different random seed than the texture of the same density used in the standard stimulus.

Image-based lighting with an infinitely distant spherical emitter was used as the sole illumination. The illumination texture was a high dynamic range image of a natural daylight outdoor scene with a partly cloudy sky. The camera settings (location and field of view) corresponded to the actual experimental setup. Thus, the stimuli appeared in nearly the same way as a corresponding real scene, except for the lack of binocular disparity. Stimuli were rendered as 16-bit high dynamic range images (“extended volumetric path tracer”; “low discrepancy sampler” with 512 samples/px; Gaussian reconstruction filter with *SD* = .5) and subsequently tonemapped to 8-bit low dynamic range images (gamma = 1.6; exposure = 1.0). The final image size was 370 × 370 px which corresponded to 100 × 100 mm on the screen.

#### Procedure

In each trial, a fixed standard and an adjustable test stimulus were presented simultaneously on an Eizo ColorEdge CG243W LCD screen (display area 518.4 × 324.0 mm; resolution 1920 × 1200 px; color depth 8-bit per channel; 3.704 px/mm; Eizo Corporation, Hakusan, Japan), which was located in a darkened room. The standard stimulus was presented in the upper half of the screen, the test stimulus in the lower half. The viewing distance was 40 cm. The subjects’ task was to adjust the test object until it appeared to be made of the same material as the fixed standard object. The refractive index of the test object was adjusted with the arrow keys of a standard computer keyboard. Each subject performed 27 trials in a pseudo-randomized order: 3 *R*_S_ × 3 *D*_S_ × 3 repetitions.

This and all following experiments were carried out in accordance with the relevant institutional and national regulations and legislation and with the World Medical Association Helsinki Declaration as revised in October 2008.

#### Subjects

Seven subjects, six of them female, participated in the experiment. Their age ranged from 20 to 27. All subjects were naïve as to the purpose of the experiment, except one, which was one of the authors (N. S.). They reported normal or corrected-to-normal visual acuity and showed no color vision deficiency, as tested by Ishihara plates (1969).

#### Results

**Figure 4.**
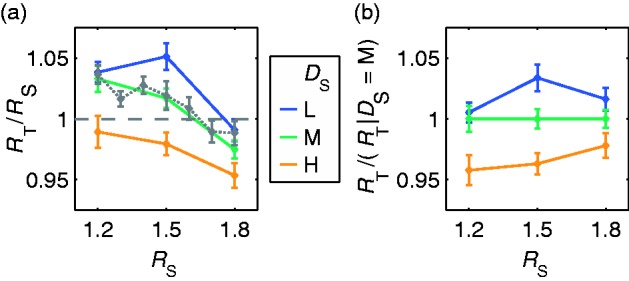
Results of Experiment 1a. (a) The adjusted refractive indices (displayed as test to standard ratios *R*_T_/*R*_S_ ± *SEM*) obtained with different background texture densities in standard and test (i.e., *D*_S_ ∈ {#x0201C;L”, “H”}) deviate systematically from those obtained with an identical texture density (i.e., *D*_S_ = “M”). The settings for trials with the same texture density deviate only marginally from those found with uniform backgrounds (dotted gray line, reprinted from Schlüter and Faul, 2014, Figure 13). (b) The same test to standard ratios plotted relative to the ones obtained with identical texture density. This highlights the systematic deviations due to density differences. In both plots, each colored solid line shows the mean refractive index setting for a particular background texture density in the standard stimulus (*D*_S_) as a function of the refractive index of the standard object (*R*_S_).

The refractive indices obtained with different background texture densities in standard and test deviate systematically from those obtained with identical texture densities (Figure 4(a)). The small deviations found with identical texture densities (i.e., *D*_S_ = “M”) are very similar to those found previously with a uniform gray background under otherwise identical conditions (cf. Schlüter and Faul, 2014, Figure 13) that are given as a dotted gray line in the figure. In Figure 4(b), the settings are plotted relative to the ones obtained with identical texture density. Relative to this condition, the adjusted refractive indices are larger for lower density in the standard (i.e., *D*_S_ = “L”) and smaller for higher density (i.e., *D*_S_ = “H”). A two-way analysis of variance revealed a significant main effect of background texture density, *F*(2, 180) = 22.33, *p* < .001.

#### Discussion

The results obtained in Experiment 1a contradict the prediction derived from the RI hypothesis. Even moderately different background densities in otherwise similar stimuli lead to significantly different refractive index settings. It is less the absolute amount of these deviations but their systematic nature that is diagnostic because systematic deviations are inexplicable if the subjects’ settings are based on the postulated distortion field. One cannot exclude the possibility that a manipulation of the texture density influences the accuracy of the distortion field estimation, but this should mainly affect the size of random errors. The random errors, however, are of comparable size in all conditions.

The settings obtained in the condition with identical texture densities in standard and test closely replicate previous results with completely uniform backgrounds (Schlüter & Faul, 2014). This suggests that the deviations from identity found in this condition are not related to the properties of the background texture but due to other factors, for instance, peculiarities in the specular reflection component resulting from the slightly different shapes of the standard and test objects.

Compared with the stimuli with a uniform background that were used in Schlüter and Faul (2014), the current stimuli contain background distortions which can, according to the RI hypothesis, also be used to estimate the refractive index. However, the addition of this cue did not only fail to improve the estimation accuracy but even impaired it. This is contrary to what is expected if the RI hypothesis holds.

### Experiment 1b: Testing the Prediction of the Image-Matching Hypothesis

The alternative explanation presented in Schlüter and Faul (2014) assumes that the subjects do not compare estimated refractive indices in such experiments but simply maximize the similarity of the presented background textures on the image-level. Such image-level matches can potentially refer to any statistic of texture attributes. An important property of this kind of match is that it is completely unrelated to transparency perception. This distinguishes it from the refractive index match assumed by Fleming et al. (2011).

**Figure 5.**
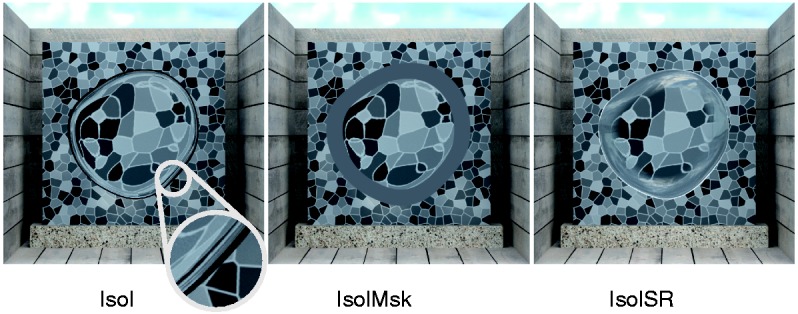
Stimulus conditions of Experiment 1b. We used the same stimuli as in Experiment 1a but isolated the influence of background distortions on the subjects’ settings. In the condition “Isol”, all specular reflections were omitted to isolate the information from background distortions. To hide rudiments of the specular reflections (black pixels at areas of former total reflections, see inset picture in the “Isol” stimulus), a ring shaped gray mask was added in the control condition “IsolMsk”. To retain the impression of a material, and thus the plausibility of the material match instruction, we added static specular reflections to the border of the object in a second control condition (“IsolSR”).

It is at present unclear which information is used in this type of match. The settings that would result under the conditions of Experiment 1a must therefore be determined empirically. To this end, we isolated the background distortions in the stimuli used in Experiment 1a by removing all specular reflections (Figure 5, condition “Isol”). After this manipulation, the transparency impression is almost completely lost. For the present purposes, this is an advantage because this makes it highly plausible that the subjects actually perform image-level matches as intended.

A problem arising in this process is that areas of total reflection persist in the image as black areas at the object’s border. To prevent subjects from matching these black ring-like areas, whose width correlates with the refractive index, we masked the border area of the object with a gray ring (Figure 5, condition “IsolMsk”).

To be able to draw conclusions from the results of this experiment on the matching behavior in Experiment 1a, we kept most methodological aspects identical, including the instruction that asked the subjects to match the objects’ material properties. We suspected that this might confuse the subjects because the material impression in the manipulated stimuli is weak or even absent. To control for this, we restored a material impression by adding a fixed specular reflection pattern to the border of the objects (Figure 5, condition “IsolSR”), which did not depend on the refractive index that was used to compute the background distortion in standard and test.

#### Stimuli

The stimuli were the same as those used in Experiment 1a, except for the following differences: For the condition “Isol”, all specular reflections were omitted during the rendering process. The resulting darkening of the object area (according to Fresnel’s equations, the amount of light that is transmitted through a surface decreases with increasing refractive index) was cancelled by correcting the average brightness of the object area to keep subjects from using it as a matching criterion. In the control condition “IsolMsk,” remnants of the specular reflections (black ring-like areas that occurred at areas of former total reflections, cf. Figure 5) were masked by a gray ring (*x* = .31, *y* = .31, *L* = 17.21 cd/m^2^). In the second control condition “IsolSR,” fixed specular reflections were added to the inner object border by superimposing an image of an object with a constant refractive index (*R* = 1.5) in front of a uniform background texture (*x* = .31, *y* = .32, *L* = 17.21 cd/m^2^). The inward transition to the original stimulus was blurred.

#### Procedure

The procedure was the same as in Experiment 1a. However, each subject performed 81 trials in a pseudo-randomized order (3 *R*_S_ × 3 *D*_S_ × 3 isolation conditions × 3 repetitions).

#### Subjects

The subjects were the same as in Experiment 1a.

#### Results

**Figure 6.**
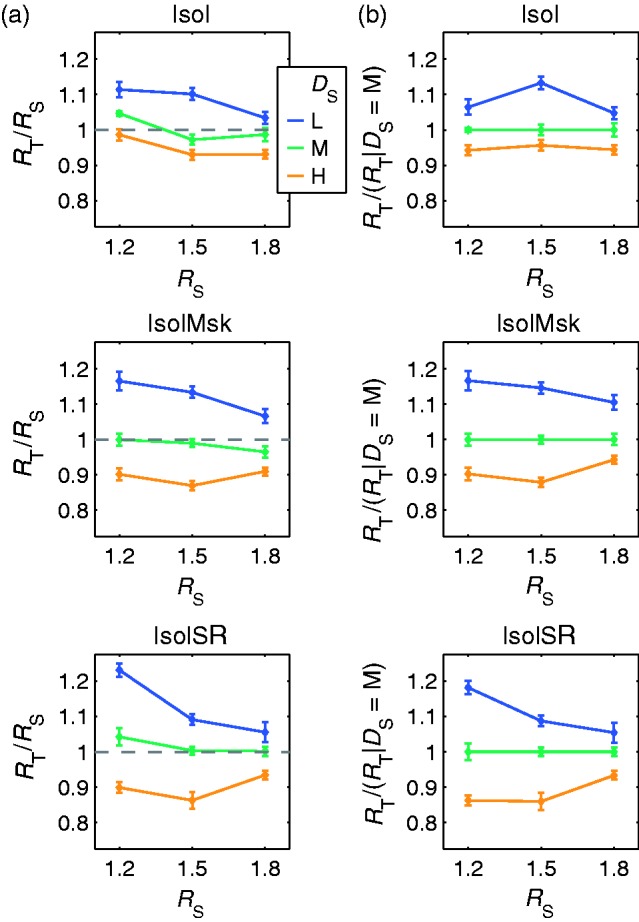
Results of Experiment 1b. (a) The refractive index settings (displayed as test to standard ratios *R*_T_/*R*_S_ ± *SEM*) obtained with different background texture densities in standard and test (i.e., *D*_S_ ∈ {#x0201C;L”, “H”}) deviate systematically from those obtained with identical texture density in standard and test (i.e., *D*_S_ = “M”). The pattern of deviations is similar in all conditions, but their size is slightly smaller in the “Isol” condition than in the two control conditions “IsolMsk” and “IsolSR”. (b) To highlight the systematic deviations that can be attributed to density differences, the same test to standard ratios are plotted relative to the ones obtained with identical density in standard and test (i.e., *D*_S_ = “M”). Each colored solid line shows the mean refractive index setting for a particular background texture density in the standard stimulus (*D*_S_) as a function of the refractive index of the standard object (*R*_S_).

For all conditions, the refractive index settings deviate systematically from those given in the standard object (Figure 6(a)). If the background texture density of the standard is lower than that of the test (i.e., *D*_S_ = “L”), the refractive index settings are higher than the given ones (*R*_T_/*R*_S_ > 1), while they are lower for a higher density in the standard (i.e., *D*_S_ = “H”). The deviations are comparatively small if standard and test have the same texture density (i.e., *D*_S_ = *D*_T_ = “M”). The size of the deviations in the two control conditions (“IsolMsk” and “IsolSR”) is similar and slightly larger than in the “Isol” condition.

**Figure 7.**
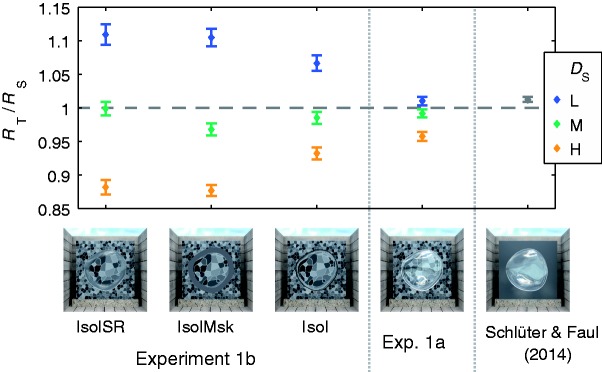
The results of Experiments 1b and 1a in comparison with the settings for a uniform background reported in Schlüter and Faul (2014). While the general pattern of deviations is similar in Experiments 1a and 1b, the deviations differ in size and are considerably larger in Experiment 1b. The settings obtained in matches of isolated specular reflections are close to identity. These results suggest that the size of the deviations decreases as the relative salience of reflection-related information increases (from “IsolSR” on the left to the uniform background on the right). Points of the same color correspond to the mean test to standard ratio (*R*_T_/*R*_S_ ± *SEM*) for a particular background texture density in the standard stimulus (*D*_S_) averaged across all refractive indices of the standard object (*R*_S_).

These differences become especially apparent if the settings are averaged across all *R*_S_ (Figure 7, left three columns). If the settings are directly compared with the ones gained in Experiment 1a (Figure 7, fourth column), it becomes apparent that the general pattern of deviations is the same in both experiments, but that the size of the deviations is much larger in Experiment 1b. The settings for stimuli with uniform backgrounds that were reported in Schlüter and Faul (2014) are depicted for comparison (see discussion).

#### Discussion

**Figure 8.**
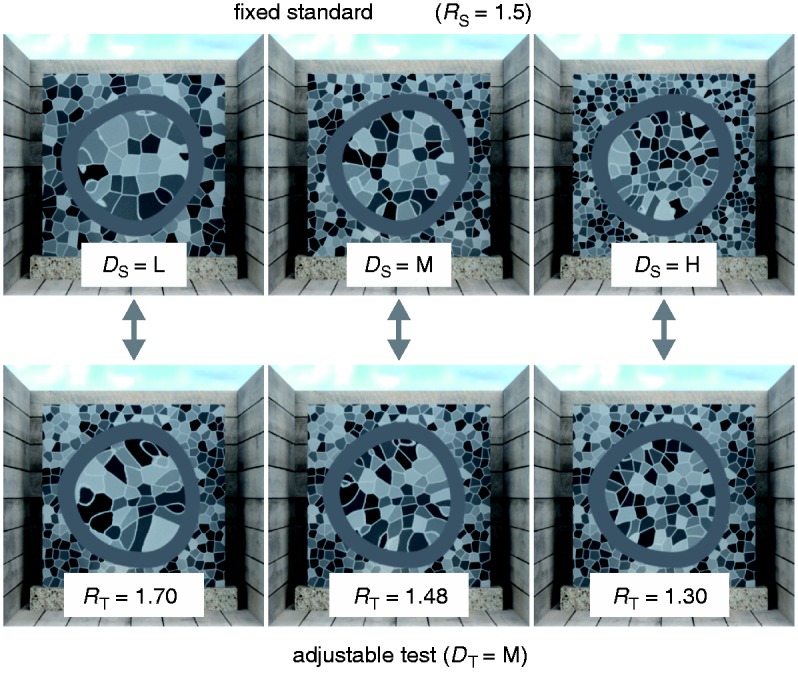
Exemplary depiction of the subjects’ mean settings in the test object (bottom row) in the control condition “IsolMsk” for standard stimuli (top row) with a fixed refractive index (*R*_S_ = 1.5) and a background texture density (*D*_S_) that was either lower (left column, “L”), the same (middle column, “M”), or higher (right column, “H”) than the one of the test stimulus (*D*_T_ = “M”). If standard and test had different background texture densities, the adjusted refractive indices (*R*_T_) differed systematically from the fixed ones given in the standard (*R*_S_). The depicted results suggest that the subjects tried to equate the average element size of the distorted background textures.

In this experiment, the subjects had only the (distorted) backgrounds available for their matches. Figure 8 shows the subjects’ mean test settings for standards with different texture densities. Comparing the background patterns suggests that the subjects matched the average element size of the distorted background across the scenes. A low background texture density in the standard results in a large average element size within the distorted background. To match this, the test’s refractive index has to be increased above the standard’s one until the magnification effect leads to a similar average element size (roughly speaking, increasing refraction increases the magnifying effect of the convex light-transmitting object, which in turn increases the average element size). Mutatis mutandis, a standard with a higher background texture density leads to the prediction of a deviation in the opposite direction.

The similarity of the pattern of deviations found in Experiments 1a and 1b suggests that image-level matches of the distorted background textures had also played a critical role in Experiment 1a. The considerably smaller size of the deviations in Experiment 1a can be explained by assuming that the subjects also used information from specular reflections. Isolated matches of specular reflections have been found to lead to settings that are almost identical to the ones given in the standard (see replot of data from Schlüter and Faul, 2014, in Figures 4 and 7). Because both types of matches require different adjustments of the refractive index, the subjects likely settled on a compromise. This would explain the shift toward identity with increasing relative salience of reflection-related information.

This interpretation is also supported by the finding that the deviations are lower in the “Isol” condition than in the two control conditions because the traces of information from specular reflections available in the “Isol” condition may also have shifted the subjects’ settings closer towards identity. The smaller degree of this shift might be explained by the plausible assumption that the “black ring” cue is less salient than specular reflections and therefore is given a lower weight in the compromise.

The similarity of the results in the “IsolSR” and the “IsolMsk” condition suggests that the subjects were not confused by the fact that we retained the material matching instruction although the impression of an object with distinct material properties is rather weak or even absent in the “Isol” and “IsolMsk” conditions.

## Experiment 2: The Role of the Surround

Experiment 2 is related to the assumption of the RI hypothesis that estimating the distortion field requires a comparison of distorted and undistorted background areas. In Experiment 2a, we tested a prediction derived from this assumption. In Experiment 2b and 2c we compare these results with predictions from our alternative explanation.

### Experiment 2a: Testing the Prediction of the RI Hypothesis

**Figure 9.**
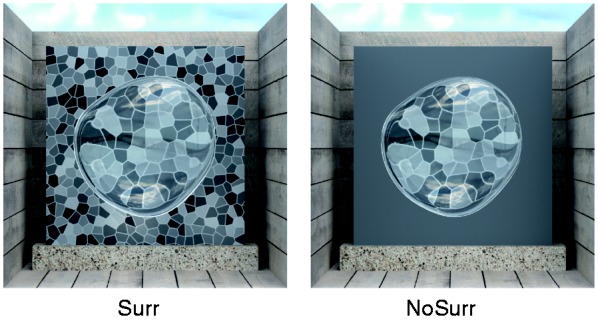
Stimulus conditions of Experiment 2a. Because estimating the distortion field presumably requires a comparison of the distorted background within the object boundaries with the undistorted background in the surround, the subjects’ settings should become highly unreliable if the surround is removed. To test this prediction, we used similar stimuli as in Experiment 1a. The surround was either left unchanged (“Surr”), or it was replaced by a uniform gray background (“NoSurr”).

Fleming et al. (2011) assume that using the distortion field as a cue “involves comparing the relative scale of texture elements seen through the transparent object with the elements seen directly” (p. 818). This implies that the estimation of the refractive index from the distortion field should be severely hampered or may even fail completely if the texture in the object’s surround is removed. As a consequence of this manipulation, one would expect highly unreliable settings and accordingly a strong increase in the size of (random) errors. Alternatively, it would also be possible that the putative distortion field cue is identified as unreliable and simply ignored. In that case, one would expect settings similar to those obtained in Schlüter and Faul (2014) for stimuli with a uniform background, that is, approximate identity of the refractive indices in standard and test. To test these predictions, we repeated Experiment 1a with an additional condition in which the texture in the surround was replaced with a uniform gray area (condition “NoSurr”, Figure 9).

The subjects’ matching behavior may depend on the order in which the stimuli with and without a textured surround are presented to them. For example, the subjects might switch to an alternative matching strategy when the textured surround disappears for the first time and stay with it for the sake of consistency even if the textured surround becomes available again in a following trial. To control for such effects, we balanced the order with which the two surround conditions were presented to the subjects. Half of the subjects had the “Surr” condition first, the other half the “NoSurr” condition (“Surr→NoSurr” vs. “NoSurr→Surr”).

#### Stimuli

The stimuli were similar to those used in Experiment 1a. Again, the background texture density of the standard stimuli was varied in three steps (*D*_S_ ∈ {#x0201C;L”, “M”, “H”}), while the test stimuli always had the same density (*D*_T_ = “M”; cf. Figure 3). The undistorted background visible in the surround of the object was either left unchanged (“Surr”) or was replaced by a uniform gray background (“NoSurr”; *x* = .31, *y* = .32, *L* = 17.21 cd/m^2^) as depicted in Figure 9.

#### Procedure

The procedure was the same as in Experiment 1a. However, each subject performed 54 trials (3 *R*_S_ × 3 *D*_S_ × 2 surround conditions × 3 repetitions). In each half (i.e., “Surr”, “NoSurr”) of the conditions “Surr→NoSurr” and “NoSurr→Surr,” the trials were presented in random order.

#### Subjects

Eight subjects, six of them female, participated in the experiment. Their age ranged from 19 to 37. All subjects were naïve as to the purpose of the experiment and had not participated in similar experiments. They reported normal or corrected-to-normal visual acuity and showed no color vision deficiency, as tested by Ishihara plates (1969).

#### Results

**Figure 10.**
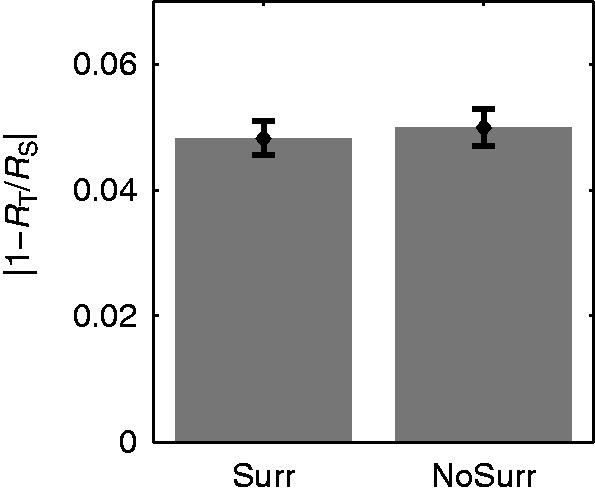
Mean absolute deviations of the adjusted refractive indices (displayed as test to standard ratios *R*_T_/*R*_S_ ± *SEM*) from identity (i.e., *R*_T_/*R*_S_ = 1) for stimuli with (“Surr”) and without (“NoSurr”) textured surround.

**Figure 11.**
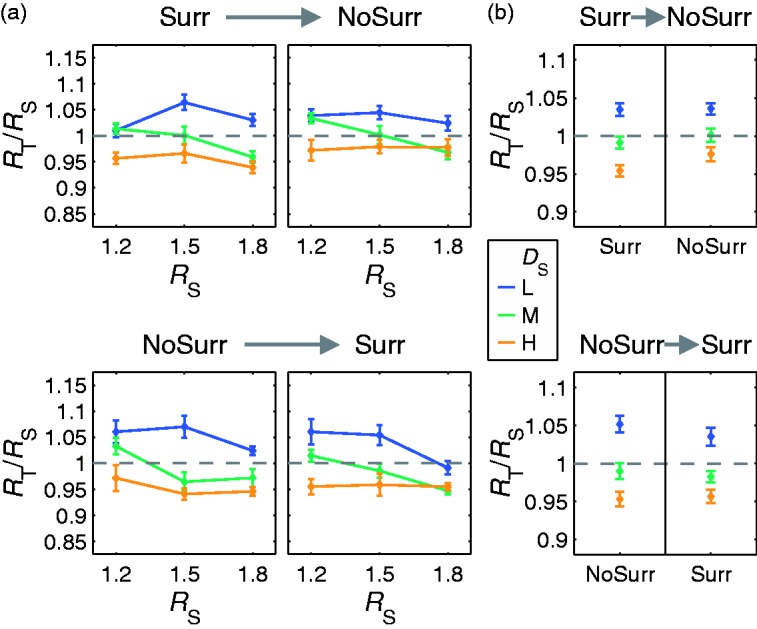
Results of Experiment 2a. Neither the availability of the surround nor the presentation order has a systematic effect on the subjects’ settings. (a) Mean refractive index setting (displayed as test to standard ratios *R*_T_/*R*_S_ ± *SEM*) for a particular background texture density in the standard stimulus (*D*_S_) as a function of the refractive index of the standard object (*R*_S_). Stimuli without a textured surround (“NoSurr”) were either shown after (“Surr→NoSurr”, top row) or before (“NoSurr→Surr”, bottom row) stimuli with a textured surround (“Surr”). (b) The same data averaged across all *R*_S_.

The subjects’ settings do *not* depend systematically on the availability of the textured surround (“Surr” vs. “NoSurr”, Figure 10) or the order by which the surround conditions were presented (“Surr→NoSurr” vs. “NoSurr→Surr”, Figure 11). The settings of the refractive indices appear almost identical to those observed in Experiment 1a. For standard stimuli with lower background texture densities (*D*_S_ = “L”), the refractive index settings are higher than the given ones (*R*_T_/*R*_S_ > 1), for those with higher background texture densities (*D*_S_ = “H”), the settings are lower (*R*_T_/*R*_S_ < 1). The deviations are considerably smaller if standard and test stimuli have the same background texture density (i.e., *D*_S_ = *D*_T_ = “M”).

#### Discussion

The prediction derived from the RI hypothesis that the errors of the refractive index settings will increase if the undistorted background texture in the surround is removed is not confirmed by our data. Instead, both the pattern of systematic deviations and the size of random errors are essentially identical for conditions with and without a textured surround. This suggests that the subjects referred solely to information within the object boundaries and ignored any information available in the surround.

Furthermore, our results show that the subjects did not ignore the remaining background texture completely, since their settings differ from those obtained with a uniform background (Schlüter & Faul, 2014).

These observations do not seem to be compatible with the computational idea underlying the RI hypothesis.

### Experiment 2b: Testing the Prediction of the Image-Matching Hypothesis

In Experiment 2b, we again isolated the background distortions to determine the settings resulting from image-level matches under the conditions realized in Experiment 2a. If the assumption is correct that the observed deviations in Experiments 1a, 1b, and 2a are the result of image-level matches of properties of the distorted textures, then the following results would be expected: (a) The *pattern of the deviations* observed in Experiments 2a and 2b should be identical because the availability of the textured surround is then irrelevant for the settings. (b) The *absolute size of the deviations* should be larger in Experiment 2b than in 2a because the compensating effect of the reflection cue is no longer available (see discussion of Experiment 1b). (c) The results of Experiments 1b and 2b should be similar because under the above assumption the two conditions were equivalent.

**Figure 12.**
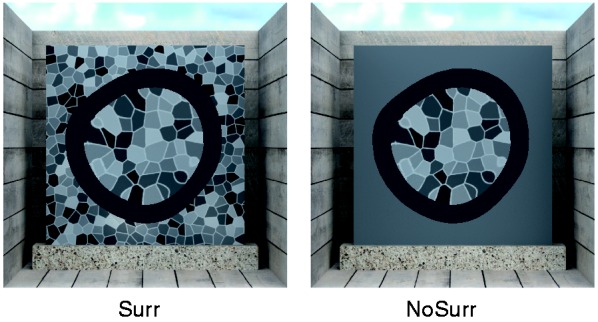
Stimulus conditions of Experiment 2b. We used the same stimuli as in Experiment 2a but isolated the influence of background distortions on the subjects’ settings. If subjects only refer to the object’s area, then the availability of the textured surround should not have an influence on their settings.

We only used a single isolation condition (“IsolMsk”, see Experiment 1b), and the surround was manipulated in the same way as in Experiment 2a (see Figure 12).

**Figure 13.**
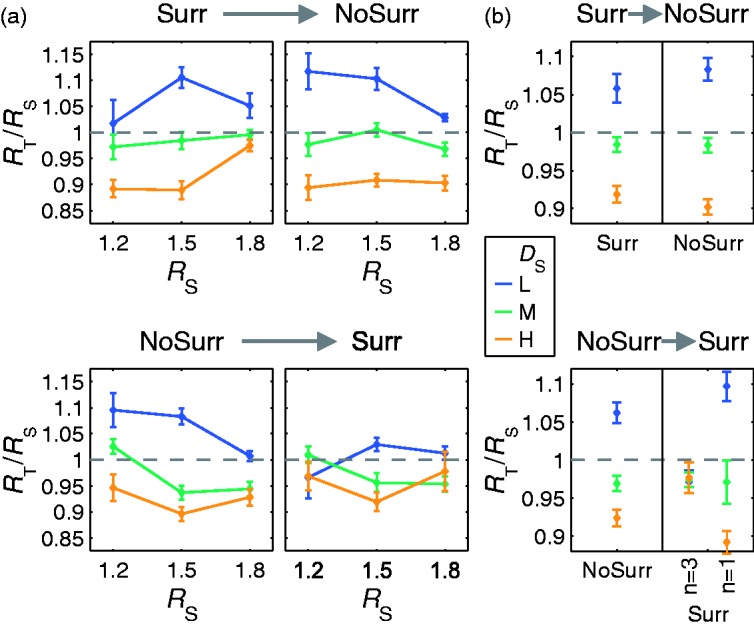
Results of Experiment 2b. The overall pattern of deviations is similar to the one found in Experiment 2a. However, if the textured surround is first shown in the second half of the experiment (“NoSurr→Surr”), then the settings almost coincide with each other, irrespective of the standard’s background texture density (*D*_S_). (a) Mean refractive index setting (displayed as test to standard ratios *R*_T_/*R*_S_ ± *SEM*) for a particular background texture density in the standard stimulus (*D*_S_) as a function of the refractive index of the standard object (*R*_S_). In the condition “Surr→NoSurr” (top row), the textured surround was shown in the first half of the experiment, in the condition “NoSurr→Surr” (bottom row) in the second half. (b) The same data averaged across all *R*_S_. The collapse of deviations for “Surr” stimuli shown in the second half of the experiment (“NoSurr→Surr”) occurs for three out of four subjects (labelled “*n* = 3”). The settings of the remaining subject are given separately (labelled “*n* = 1”). Please note that the panel on the left corresponding to this condition shows the mean across all four subjects.

#### Stimuli

The stimuli were identical to those used in Experiment 2a, except that background distortions were isolated in the same way as in the “IsolMsk” condition of Experiment 1b, that is, any specular reflections were omitted during the rendering, the brightness of the object area was corrected for any refraction-dependent darkening, and a gray mask was added to the object boundary (*x* = .31, *y* = .32, *L* = 7.88 cd/m^2^).

#### Procedure

The procedure was the same as in Experiment 2a. Again, each subject performed 54 trials (3 *R*_S_ × 3 *D*_S_ × 2 surround conditions × 3 repetitions).

#### Subjects

The subjects were the same as in Experiment 2a.

#### Results

The results of Experiment 2b are shown in Figure 13(a) and (b). Overall, the settings in trials with different texture densities in standard and test deviated from identity in a similar way as in Experiment 1b, as expected. However, a completely different pattern of results is observed in the second half of the ”NoSurr→Surr” condition: for three out of four subjects the deviations almost vanish and the refractive index settings virtually coincide with each other, irrespective of the actual background texture density. For one of the subjects, this collapse does not occur. The results of this subject are displayed separately in Figure 13(b) (labelled “*n* = 1”).

#### Discussion

In general, the pattern of deviations in this experiment is very similar to the one found in Experiment 2a. This suggests that image-level matches of background distortions had also played a critical role in Experiment 2a.

An unexpected observation is the “collapse of the deviations” in the second half of the “NoSurr→Surr” condition. These settings are close to identity and one may therefore be tempted to interpret this as support for the RI hypothesis. However, several observations speak against this view: First, large systematic deviations from identity were found if the exact same stimuli were presented at the beginning of the experiment in the “Surr→NoSurr” condition. Second, the collapse of deviations was found in a physically inconsistent situation, in which the “objects” did not even appear transparent, whereas in Experiment 2a, in which the stimuli contained at least two consistent and physically correct cues and appeared transparent, no such collapse was observed. Third, not all subjects showed this collapse of deviations.

A more plausible explanation is that the subjects in this case matched *relations* between image attributes, for instance, the ratio of the mean element size within and outside of the object’s area, instead of image attributes within the object’s boundary directly, as in the other conditions. In the particular situation realized in the experiment, this can lead to settings near identity because these relations are presumably almost invariant against changes in the background texture density. The observed result is to be expected if three of the four subjects had deliberately switched their matching strategy from absolute to relational, when the surround suddenly changed from uniform to textured in the second half of the experiment. It is at present unclear, why such a switch did not occur in Experiment 2a, but it seems plausible to assume that the salient specular reflections that also influence the settings kept the subjects focussed on the object areas.

### Experiment 2c: Testing Relational Image-Level Matches

To test, whether the collapse of deviations in Experiment 2b could be explained by a match of relations between image-attributes, we explicitly instructed the subjects in a similar matching experiment to perform this kind of match.

We also tried to address the question, whether such relational matches could constitute a viable strategy (in the sense of Fleming et al., 2011) to reliably estimate the refractive index of transparent objects. For this to be the case, the estimates need to be robust against changes in context. To test this, we varied the thickness of the standard object, that is, a context factor unrelated to material properties that also influences background distortions.

#### Stimuli

The stimuli were similar to the ones used in Experiment 2b. However, the thickness of the standard object was varied in three steps (*T*_S_ ∈ {3 mm, 6 mm, 9 mm}), while the thickness of the test object remained constant (*T*_T_ = 6 mm). Different thicknesses were implemented by applying a directional scaling factor to the meshes of the objects. In contrast to Experiment 2b, only stimuli with a textured surround (“Surr”) were used, for which the “collapse of deviations” were found.

#### Procedure

The procedure was the same as in Experiment 2b. However, each subject performed 81 trials in randomized order (3 *R*_S_ × 3 *D*_S_ × 3 *T*_S_ × 3 repetitions). The subjects were explicitly instructed to refer with their matches to the relations of distorted and undistorted background areas (“Your task is to adjust the test stimulus until the size ratio of the texture elements within the object area and its surround is the same as in the standard stimulus.”).

#### Subjects

Eight subjects, six of them female, participated in the experiment. Their age ranged from 21 to 29. All subjects were naïve as to the purpose of the experiment and did not participate in the previous experiments. They reported normal or corrected-to-normal visual acuity, and showed no color vision deficiency, as tested by Ishihara plates (1969).

#### Results

**Figure 14.**
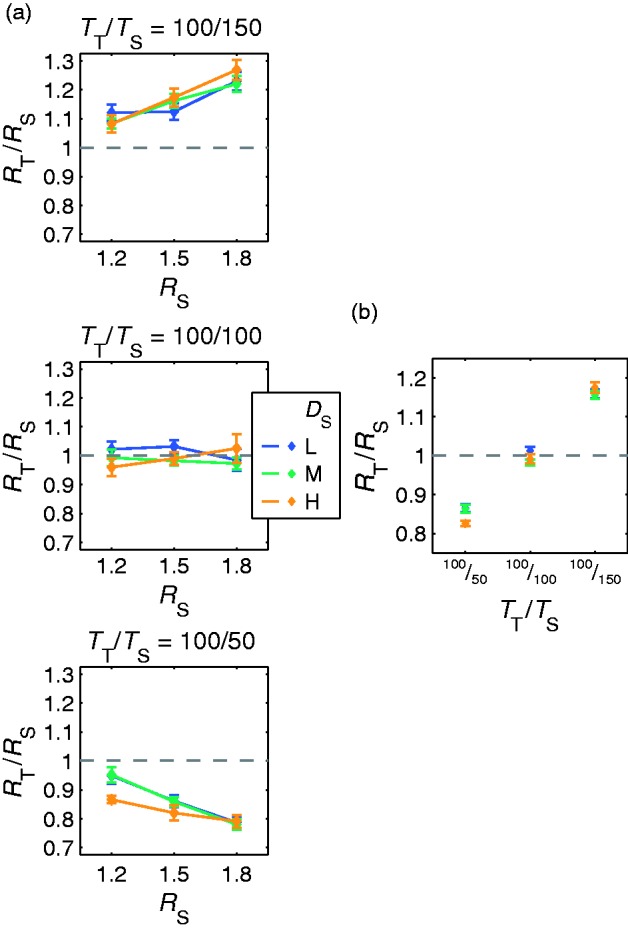
Results of Experiment 2c in which the subjects were instructed to match the relations of distorted to undistorted background areas. The refractive index settings (displayed as test to standard ratios *R*_T_/*R*_S_ ± *SEM*) depend on the object thickness (*T*_S_) but not on the texture density (*D*_S_) in the standard. (a) Each plot corresponds to one thickness of the standard object. Data points of same color show the mean refractive index settings for a particular background texture density in the standard stimulus (*D*_S_) as a function of the refractive index of the standard object (*R*_S_). (b) The same data averaged across all *R*_S_.

The results of Experiment 2c are shown in Figure 14(a) and (b). For all object thickness combinations, the settings do not depend on the background texture density. However, the settings are only near identity if standard and test objects have the same thickness. If the objects’ thickness differs, the settings deviate systematically.

#### Discussion

The settings for objects of equal thicknesses resemble the “collapse of deviations” found in Experiment 2b. This suggests that the subjects’ matching behavior was identical, despite the difference in the instructions. A plausible and parsimonious explanation of this finding is that the subjects in both experiments matched relations of texture properties between the distorted and undistorted background. An inspection of the match results suggests that the subjects referred to simple image attributes like the average element size of the background pattern. The ratio of such attributes taken between object area and surround is approximately invariant against changes in the overall background texture density. This explains the insensitivity of relational matches to differences in background texture density. Changes in the thickness of the object, in contrast, influence only the attributes within the object’s boundaries (roughly speaking, increasing the thickness of the convex object increases its magnifying effect, which in turn increases the average element size in the background area seen through the object). This explains the systematic deviations found with objects of different thicknesses. These latter results are in conflict with the assumption that referring to the relation of simple image criteria is generally sufficient to estimate the distortion field and the refractive index.

## Summary and General Discussion

In this article, we tested two predictions derived from the RI hypothesis of Fleming et al. (2011), which essentially states that the visual system can use image distortions caused by thick transparent objects to estimate the refractive index of their material. Fleming et al. (2011) propose that this estimation process comprises two stages. In the first stage, a distortion field is estimated from the input pattern, and in the second stage, the information contained in this mid-level cue is “pooled into a global estimate of refractive index” (p. 818). The exact processes were largely left unspecified by the authors. However, it is thought that the estimated distortion field approximates some important aspects of the divergence of the displacement field, which describes the actual optical distortion. It is obvious that any kind of approximation would be impossible without some information about the undistorted background. Fleming et al. (2011) therefore assume that the estimation of the distortion field “involves comparing the relative scale of texture elements seen through the transparent object with the elements seen directly” (p. 818).

Our strategy to test this computational idea was to derive predictions from it that could be tested without any knowledge about the exact nature of the putative processes. A first prediction is that the refractive index settings in a material matching task should not be influenced by isolated changes of a scene variable that is unrelated to the refractive index and does neither alter the relative scale of the texture elements inside the object and the surround nor the distortion field. In Experiment 1a, we tested this by asking subjects to match the material of two transparent objects across scenes that were identical apart from the density of their background texture. We found that even relatively small density differences had a highly *systematic* and statistically significant effect on the subjects’ settings. Fleming et al. (2011), in contrast, varied context factors (i.e., background distance and object thickness) that systematically influenced *both* the distortion field and the relative scale of texture elements within the object and its surround. The observed systematic deviations from a perfect match may thus be explained by assuming that the visual system cannot completely distinguish between different influences on the distortion field. Our strategy to keep the distortion field and the relative scale of texture elements constant excludes this kind of explanation and therefore suggests that the deviations observed in Experiment 1a are not due to a bias in the estimation of the refractive index, but that the matches were performed without referring to the distortion field at all.

The idea that the estimation of the distortion field involves a comparison of texture elements seen through the transparent object with elements seen directly leads to the further expectation that the uncertainty of refractive index settings that are based on this cue should increase sharply, if information about the undistorted background is removed. Contrary to this prediction, we found in Experiment 2a that the refractive index settings did *not* change when the texture in the surround was replaced by a uniform gray surface. Instead, we observed the same systematic deviations as in Experiment 1a. This suggests that the surround was completely ignored and that a comparison of distorted and undistorted background areas, as it has been proposed by Fleming at al. (2011), did not take place. In general, it seems impossible to estimate the distortion field without using the surround as a reference for the undistorted state of the background texture. While some information about the undistorted state may be inferred if rather strong regularity assumptions with respect to possible background patterns are made, for example, that they only contain straight lines, it would still be impossible to determine the *absolute* amount of the distortion. Such relative distortion information is insufficient to estimate the *absolute* value of the refractive index that uniquely characterizes a light-transmitting material. However, relative distortion information might be useful for other purposes, for example, to infer shape properties. Here, regions of relative magnification and compressions might serve as cues for convex or concave surface curvatures.

The results of Experiments 1a and 2a are clearly not in line with basic assumptions underlying the general computational idea outlined earlier. In three additional experiments, we tested whether the pattern of results found in Experiments 1a and 2a is compatible with simple image-level matches of the distorted background texture. The procedures and stimuli used in Experiment 1b and 2b were essentially identical to those in Experiment 1a and 2a, respectively. The sole difference was that we removed specular reflections and in this way isolated the information contained in the background distortions. This manipulation had the side effect that the “objects” did no longer appear transparent, which is an advantage in that it increases the plausibility that the subjects actually performed image-level matches. In these experiments, we observed deviations from identity that are larger in absolute value but exhibit the same pattern as in the corresponding experiments with physically plausible transparent objects. This is compatible with the interpretation that such image-level matches were also made in Experiments 1a and 2a, but that the presence of specular reflections shifted the settings closer towards identity. In Experiment 2b, we unexpectedly found “perfect matches” in a condition, where a textured surround first appeared in the second half of the experiment. The conjecture that this resulted from image-level matches of the *relation of texture elements* within and outside the objects’ boundary was confirmed in Experiment 2c. The results obtained in this experiment further indicate that such relational image-level matches are in general not a viable strategy to estimate refractive indices.

### Conclusions

The results obtained in Experiments 1a and 2a seem not compatible with fundamental principles postulated in the RI hypothesis and thus confirm the results and conclusions reported in Schlüter and Faul (2014). All results obtained so far in putative material matches of thick transparent objects can be more parsimoniously explained by the assumption that the subjects maximized the perceptual similarity of the presented stimuli on the image-level. They appear to use image criteria that vary with the adjustable refractive index parameter (e.g., the average element size of the background areas, the brightness of specular reflections, or the width of black artefacts). In the context of transparency perception, we consider such image-level matches as a methodological artefact that has no theoretical meaning. Therefore, we have not examined in detail to which image criteria the subjects actually referred. (This question might however be of interest in the field of texture perception, e.g., Balas, 2006; Beck, 1983; Bergen & Adelson, 1988; Landy & Graham, 2004; Rosenholtz, 2015.) If our interpretation is correct, then it is to be expected that the subjects will refer to other image criteria in other kinds of stimuli and that they will be unable to perform reliable matches, if they fail to find an image property that systematically varies with the adjusted parameter.

Our findings seem also instructive from a methodological point of view because they highlight potential pitfalls in “direct” tests of hypotheses. The available evidence suggests that subjects in the material matching task of Fleming et al. (2011) are unable to do what they are asked to do because the visual system cannot estimate the refractive index from image distortions in that situation. The important point here is that they do not refuse to perform a match, but instead tacitly switch to an unintended alternative task. Unfortunately, such a tacit change in task is notoriously difficult to detect because the resulting data are often highly reliable and may even partly be in line with the experimenter’s expectations. The experiment of Fleming et al. (2011) is a point in case because simple image-level matches of background distortions or specular reflections can lead to high correlations between the refractive index settings and the refractive index given in the standard and this can easily be mistaken as the result of refractive index matches. The mere fact that subjects were presented with transparent objects, adjusted refractive indices and were asked to match the objects’ material does not guarantee that conclusions about transparency perception can be drawn from the obtained results.

### Outlook

The theoretical considerations and empirical findings presented in Schlüter and Faul (2014) and the present article strongly suggest that observers are unable to estimate the refractive index from optical distortions in experimental settings similar to the ones realized by Fleming et al. (2011). Strictly speaking, this conclusion is limited to the situations realized in these investigations. One may argue that the stimuli used in these experiments did not contain enough information to estimate and match the objects’ refractive indices and that this forced the subjects to perform image-level matches. This hypothesis can—and should—be tested with more complex stimuli that provide additional information, for example, stereo and motion cues that may support the estimation of the distortion field and that facilitate the adequate consideration of irrelevant context factors. Unfortunately, such investigation will also be confronted with the difficult problem of how to control for the effects of specular reflections and other potential information related to the refractive index that may be available in more complex scenes. Simply removing additional information seems problematic because this may reduce or even destroy the transparency impression. This was unproblematic in our Experiments 1b and 2b but is clearly undesirable if one tries to find support for the RI hypothesis. Even if it is possible to discard secondary cues without destroying the impression of transparency, this could nevertheless change the role the (isolated) cue of interest plays, depending on how the visual system actually integrates different cues (cf. Ernst & Bülthoff, 2004; Landy, Maloney, Johnston, & Young, 1995).

Whether the RI hypothesis can be confirmed in more complex scenes is an open empirical question. However, on theoretical grounds, we are not very optimistic about a positive outcome. In Schlüter and Faul (2014), we mentioned several reasons why we think that the RI hypothesis is a priori implausible. Apart from serious computational difficulties inherent in estimating refractive indices from background distortions, it remains unclear of what use such estimates could be, especially because it is seems probable that they cannot be very reliable.

A serious problem for tests of the RI hypothesis in its present form is that the specification of putative mechanisms is rather vague. It would be desirable to develop proposals that are more specific and to investigate with the help of computer simulations, whether and, if so, under which conditions they are sufficient to solve the problem of estimating the refractive index.

## References

[bibr1-2041669516669616] Balas B. J. (2006) Texture synthesis and perception: Using computational models to study texture representations in the human visual system. Vision Research 46: 299–309. doi:10.1016/j.visres.2005.04.013. 15964047 10.1016/j.visres.2005.04.013

[bibr2-2041669516669616] Beck J. (1978) Additive and subtractive color mixture in color transparency. Perception & Psychophysics 23: 265–267. doi:10.3758/BF03204137. 662577 10.3758/bf03204137

[bibr3-2041669516669616] Beck J. (1983) Textural segmentation, second-order statistics, and textural elements. Biological Cybernetics 48: 125–130. doi:10.1007/BF00344396. 6626590 10.1007/BF00344396

[bibr4-2041669516669616] Beck J. Prazdny K. Ivry R. (1984) The perception of transparency with achromatic colors. Perception & Psychophysics 35: 407–422. doi:10.3758/BF03203917. 6462867 10.3758/bf03203917

[bibr5-2041669516669616] Bergen J. R. Adelson E. H. (1988) Early vision and texture perception. Nature 333: 363–364. doi:10.1038/333363a0. 3374569 10.1038/333363a0

[bibr6-2041669516669616] Blake A. Bülthoff H. (1990) Does the brain know the physics of specular reflection? Nature 343: 165–168. doi:10.1038/343165a0. 2296307 10.1038/343165a0

[bibr7-2041669516669616] Blake A. Bülthoff H. (1991) Shape from specularities: Computation and psychophysics. Philosophical Transactions of the Royal Society B: Biological Sciences 331: 237–252. doi:10.1098/rstb.1991.0012. 10.1098/rstb.1991.0012 1674154

[bibr8-2041669516669616] Blender Foundation. (2013). *Blender—A 3D modelling and rendering package* (Version 2.66a). Retrieved from http://www.blender.org.

[bibr9-2041669516669616] Chen J. Allison R. S. (2013) Shape perception of thin transparent objects with stereoscopic viewing. ACM Transactions on Applied Perception 10: 1–15. doi:10.1145/2506206.2506208.

[bibr10-2041669516669616] Ernst M. O. Bülthoff H. H. (2004) Merging the senses into a robust percept. Trends in Cognitive Sciences 8: 162–169. doi:10.1016/j.tics.2004.02.002. 15050512 10.1016/j.tics.2004.02.002

[bibr11-2041669516669616] Faul F. Ekroll V. (2002) Psychophysical model of chromatic perceptual transparency based on substractive color mixture. Journal of the Optical Society of America. A, Optics, Image Science, and Vision 19: 1084–1095. 10.1364/josaa.19.001084 12049345

[bibr12-2041669516669616] Faul F. Ekroll V. (2011) On the filter approach to perceptual transparency. Journal of Vision 11: 1–33. doi:10.1167/11.7.7. 10.1167/11.7.7 21659428

[bibr13-2041669516669616] Fleming R. W. Bülthoff H. H. (2005) Low-level image cues in the perception of translucent materials. ACM Transactions on Applied Perception 2: 346–382. doi:10.1145/1077399.1077409.

[bibr14-2041669516669616] Fleming R. W. Jäkel F. Maloney L. T. (2011) Visual perception of thick transparent materials. Psychological Science 22: 812–820. doi:10.1177/0956797611408734. 21597102 10.1177/0956797611408734

[bibr15-2041669516669616] Fleming R. W. Torralba A. Adelson E. H. (2004) Specular reflections and the perception of shape. Journal of Vision 4: 798–820. doi:10.1167/4.9.10. 15493971 10.1167/4.9.10

[bibr16-2041669516669616] Ishihara S. (1969) Tests for color blindness, Tokyo: Kanehara Shuppan Co. Ltd.

[bibr17-2041669516669616] Jakob, W. (2013). *Mitsuba rendering system* (Version 0.4.4). Retrieved from http://www.mitsuba-renderer.org.

[bibr18-2041669516669616] Khang B. G. Zaidi Q. (2002a) Accuracy of color scission for spectral transparencies. Journal of Vision 2: 451–466. doi:10.1167/2.6.3. 12678644 10.1167/2.6.3

[bibr19-2041669516669616] Khang B. G. Zaidi Q. (2002b) Cues and strategies for color constancy: Perceptual scission, image junctions and transformational color matching. Vision Research 42: 211–226. doi:10.1016/S0042-6989(01)00252-8. 11809474 10.1016/s0042-6989(01)00252-8

[bibr20-2041669516669616] Landy M. S. Graham N. (2004) Visual perception of texture. In: Werner J. S. Chalupa L. M. (eds) The visual neurosciences, Cambridge, MA: MIT Press, pp. 1106–1118.

[bibr21-2041669516669616] Landy M. S. Maloney L. T. Johnston E. B. Young M. (1995) Measurement and modeling of depth cue combination: in defense of weak fusion. Vision Research 35: 389–412. doi:10.1016/0042-6989(94)00176-M. 7892735 10.1016/0042-6989(94)00176-m

[bibr22-2041669516669616] The MathWorks, Inc. (2013) *MATLAB and Statistics Toolbox Release 2013a*. Natick, MA.

[bibr23-2041669516669616] Motoyoshi I. (2010) Highlight-shading relationship as a cue for the perception of translucent and transparent materials. Journal of Vision 10: 1–11 doi:10.1167/10.9.6. 10.1167/10.9.6 20884604

[bibr24-2041669516669616] Ripamonti C. Westland S. Da Pos O. (2004) Conditions for perceptual transparency. Journal of Electronic Imaging 13: 29–35. doi:10.1117/1.1636764.

[bibr25-2041669516669616] Robilotto R. Khang B. G. Zaidi Q. (2002) Sensory and physical determinants of perceived achromatic transparency. Journal of Vision 2: 388–403. doi:10.1167/2.5.3. 12678653 10.1167/2.5.3

[bibr26-2041669516669616] Rosenholtz R. (2015) Texture perception. In: Wagemans J. (ed.) Oxford handbook of perceptual organization, Oxford, England: Oxford University Press.

[bibr27-2041669516669616] Savarese S. Chen M. Perona P. (2004) Recovering local shape of a mirror surface from reflection of a regular grid. In: Pajdla T. Matas J. (eds) Computer vision—ECCV 2004 Vol. 3023, Berlin, Germany: Springer, pp. 468–481.

[bibr28-2041669516669616] Savarese S. Chen M. Perona P. (2005) Local shape from mirror reflections. International Journal of Computer Vision 64: 31–67. doi:10.1007/s11263-005-1086-x.

[bibr29-2041669516669616] Savarese S. Fei-Fei L. Perona P. (2004) What do reflections tell us about the shape of a mirror? Proceedings of the 1st Symposium on Applied Perception in Graphics and Visualization, New York, NY: ACM, pp. 115–118. doi:10.1145/1012551.1012571.

[bibr30-2041669516669616] Schlüter N. Faul F. (2014) Are optical distortions used as a cue for material properties of thick transparent objects? Journal of Vision 14: 1–14. doi:10.1167/14.14.2. 10.1167/14.14.2 25476707

